# Effect of Immersion Time of Chicken Breast in Potato Starch Coating Containing Lysine on PhIP Levels

**DOI:** 10.3390/foods13020222

**Published:** 2024-01-10

**Authors:** Parastou Farshi, Jayendra Amamcharla, Kelly Getty, J. Scott Smith

**Affiliations:** Food Science Institute, Kansas State University, Manhattan, KS 66506, USA; pfarshi@ksu.edu (P.F.); jayendra@ksu.edu (J.A.); kgetty@ksu.edu (K.G.)

**Keywords:** chicken, coating, starch, lysine, PhIP

## Abstract

This study investigated the effect of immersion time of chicken breasts in potato starch (PS) coating containing amino acids (AAs) on the formation of 2-amino-1-methyl-6-phenylimidazo [4,5-b] pyridine (PhIP) and to evaluate a possible mechanism to inhibit the formation of PhIP in chicken breasts during frying. The chicken breasts with standardized dimensions were dipped in the potato starch (PS) coating solution containing 0.25% *w/v* lysine (Lys) for different times (15 min, 30 min, 1 h, 3 h, and 6 h). After drying the coating on the chickens, samples were fried at 195 °C for 7.5 min on each side. Results showed that the immersion time does not significantly decrease (*p* < 0.05) the PhIP level, suggesting that 15 min immersion time is enough for PhIP reduction compared to the control chicken samples (without coating). Phenylacetaldehyde (PheAce) was increased in chicken breast coated with PS-0.25% Lys after frying, suggesting that there should be another pathway to prevent the formation of PhIP by the addition of PS-0.25% Lys. Volatile compound analysis also confirmed this and showed increases in many aroma compounds in the coated chicken. Moreover, no significant differences (*p* < 0.05) were shown between the cooking loss percentage, color parameters, texture profile, and tenderness of chicken with the PS-0.25% coating and chicken without coating.

## 1. Introduction

Heterocyclic amines (HAs) are known as genotoxic carcinogens which are associated with important types of human cancer such as breast, colon, or pancreatic cancers among people consuming meat [1]. The type of meat, temperature, time of cooking, and muscle quality have been shown to affect the formation of HAs in meats [2,3,4]. The most abundant of these compounds is 2-Amino-1-methyl-6-phenylimidazo [4,5-b]-pyridine (PhIP) with a usual range of 0.3 to 182 ng/g, especially in poultry [5,6]. Due to the strong mutagenic properties of PhIP, this HCA was regarded as a possible class 2B human carcinogen by the International Agency for Research on Cancer (IARC) and was anticipated to be a human carcinogen by the National Toxicology Program (NTP) in its 12th report [7]. Therefore, investigating methods to prevent the formation of PhIP is critical. Amino acids (AAs) have been extensively used in commercial foods as nutritional supplements and have been shown to significantly reduce the level of HAs [8,9]. Among many polysaccharides studied to develop edible coatings, potato starch (PS) is an excellent film-former. It is biocompatible, biodegradable, easily accessible, and a cost-effective compound [10]. Moreover, coatings based on starch are appropriate for use in new applications through the incorporation of additional ingredients into their formulation [11]. Lys was previously shown to decrease the PhIP either by direct addition to beef patties [9] or by incorporation in edible films that were applied to the chicken as a coating before frying [12]. Even low concentrations of Lys (0.25%) could decrease PhIP levels up to 90% compared to the control chicken without coating [12]. However, to our knowledge, no study has shown the effect of AA incorporation time on PhIP levels.

Moreover, in previous studies, the possible PhIP prevention mechanisms of AAs in model systems were reported to be the formation of AA-PhIP [8], AA- phenylacetaldehyde (PheAce) adducts, or AA-benzaldehyde adducts [13]. The Strecker aldehyde of phenylalanine, PheAce (the Strecker aldehyde of phenylalanine), was reported to be an important intermediate in PhIP formation [13]. However, there is no study on the possible prevention mechanisms in chickens. In this study, we aimed to investigate the effect of immersion of chicken breast in a PS coating containing 0.25% Lys (PS-0.25% Lys) for different times (15 min, 30 min, 1 h, 3 h, and 6 h) and the coating’s effect on the PhIP level, PheAce content, volatile compounds, color, and texture of the chicken compared to the control chicken breast without coating.

## 2. Materials and Methods

### 2.1. Chemicals and Materials

The 2-amino-1-methyl-6-phenylimidazo [4,5-b] pyridine (PhIP) standard was purchased from Cayman Chemical (Ann Arbor, MI, USA). The L-lysine (Lys), glycerol (GLY), triethylamine, acetic acid (HPLC grade), formic acid, methanol, acetonitrile (HPLC grade), and ammonium acetate were purchased from Millipore Sigma (Burlington, MA, USA). Deionized water was obtained from a Sybron/Barnstead PCS filtration unit (Barnstead/Thermolyne, Inc, Dubuque, IA, USA). Bond Elut Enhanced Matrix Removal (EMR)-Lipid (QUEChERS dSPE EMR and Lipid-Polish) was obtained from Agilent Technologies (Santa Clara, CA, USA). Chicken breasts (Smart Chicken breast), potato starch (PS), and bamboo skewers were obtained from a local store. PTFE/red rubber septa was purchased from Sigma-Aldrich (St. Louis, MO, USA). A 75 µm carboxen/polydimethylsiloxane (CAR/PDMS) SPME fiber was ordered from Supelco™ Inc. (St. Louis, MO, USA).

### 2.2. Edible Coating Preparation

The PS coating solution was prepared based on a previous study with minor modifications [14]. Potato starch powder (5 g) was dissolved in 100 mL distilled deionized H_2_O, and subsequently, the solution was heated to 80 °C for 30 min for gelatinization. After cooling the solution at room temperature, 2 g/100 g (*w/w*, on a dry basis of the weight of starch) GLY (plasticizer) and 0.25 g Lys (*w/v* of the solution) were added to the solution and the resulting dispersion was mixed for 5 min. The selection of concentrations was based on the results of a previous study by authors [15]. A coating solution without AA was used for comparison. The casting solution was then homogenized at 11,000 rpm for 5 min using a laboratory homogenizer (Homogenizer, OMNI International, Kenneswa, GA, USA), and the solution was used for immersion of chicken breasts before frying.

### 2.3. Treatment of Chicken Breasts

Fresh chicken breasts were kept at refrigerator temperature (4 °C) before application of the coating solutions. Chicken breast cuts with the same dimensions (5 × 2 × 1.5 cm) were dipped in the PS coating solutions containing 0.25% Lys and were kept at 4 °C for 15 min, 30 min, 1 h, 3 h, and 6 h. Chicken breasts with no coating were used as control for comparison. Chicken breasts were then removed and after draining the excess coating, they were put on bamboo skewers under a biological containment hood for 20 min. The chicken breasts were then fried on an electrical frying pan with a surface temperature of 195 °C for 7.5 min on each side (in peanut oil). The chicken breasts were cooled at room temperature and used for further analysis.

### 2.4. PhIP Determination

PhIP extraction: Enhanced Matrix Removal-Lipid method.

The PhIP extraction was performed using the Agilent Bond Enhance Matrix Removal-Lipid (EMR-Lipid) method according to a study [16]. A finely ground 3 g sample of fried chicken breast crust was put in a 50 mL polypropylene (PP) centrifuge tube and mixed with 15 mL of formic acid (2%) in an acetonitrile solution on a mechanical shaker at room temperature for 30 min. The tube was centrifuged (10 min, 10,000× *g* at 4 °C). Subsequently, 1 g of EMR-Lipid dSPE sorbent, which was preactivated with 5 mL of 5 mM ammonium acetate buffer, was added to 12 mL of the supernatant from the centrifuge and the tubes were shaken for 2 min and centrifuged with the same conditions. Later, for a salting-out extraction and further cleanup, 10 mL of supernatant was added to the tubes containing 2 g EMR-Lipid polish salts and the tubes were shaken for 2 min and centrifuged with the same conditions. Then, from the final supernatant, 1 mL was taken to a conical vial and was dried completely under a constant flow of nitrogen gas. After drying, 100 µL of methanol was added to the vial (for reconcentration) and the final solution was filtered through a 0.2 µm nylon filter before HPLC analysis. The recovery of this extraction method was reported to be 80%, according to a previous study [12].

### 2.5. Identification and Quantification of PhIP

The PhIP was detected by HPLC (Agilent Technologies, 1100 series) coupled with a fluorescence detector (Hewlett-Packard, Palo Alto, CA, USA) according to a method described by [17] with some modifications. The emission and excitation wavelengths of the detector were 306 and 371 nm, and the column was C18 (TOSOH Bioscience LC, King of Prussia, PA, USA, TSKgel ods-80, 4.6 mm ID × 25 cm, 5 micrometer Millipore Sigma (Burlington MA, USA). The gradient mobile phase consisted of acetonitrile (A) and 0.01 M triethylamine solution (B) which was adjusted to pH = 3.6 using acetic acid. The mobile phase started with 20% A to 80% B, and it was programmed to 25% A to 75% B for 4 min, and 55% A to 45% B for 20 min. The flow rate was 1.0 mL/min during the run time. The injection volume was 10 μL and the column temperature was 26 °C. The matrix-matched standard curve was used for the calculation of PhIP concentration (ng per g of fried chicken breast crust). The PhIP percent reduction was calculated based on the following equation:

Percent Reduction = (Cc − Cs/Cc) × 100, where Cc was the PhIP concentration of the control chicken breast without coating and Cs was the PhIP concentration of chicken breast dipped in PS coating.

### 2.6. Extraction of Phenylacetaldehyde

Phenylacetaldehyde extraction was conducted with the method described by Cheng et al. [18]. The ground chicken crust (5 g) was mixed with ethyl acetate (20 mL). The mixture was shaken and subjected to ultrasonic extraction for 10 min. After static stratification, the ethyl acetate phase was collected and this extraction method was repeated twice. The ethyl acetate extraction layers were combined and the sample was dried under nitrogen gas flow. Then, 25 mL methanol was added for reconcentration and 1 µL of it was taken with a syringe for injection.

### 2.7. Extraction of Volatile Compounds

Chicken crust (1.5 g) was transferred to screw cap GC vials (20 mL thread vial and 1.3 mm caps) and sealed with PTFE/red rubber septa. A 75 µm carboxen/polydimethylsiloxane (CAR/PDMS) SPME fiber from Supelco™ Inc. (St. Louis, MO, USA) was used. The samples were heated at 70 °C for 20 min in an aluminum heating block to pre-equilibrate the headspace. The CAR/PDMS SPME fiber was exposed in the upper space of the vial for 40 min to adsorb volatiles in the headspace. Then, the fiber was withdrawn into a manual holder and manually injected into the GC injector. The fiber was kept for 5 min in the injector for desorption.

### 2.8. Volatile Compound Analysis by GC–MS

The GC–MS chromatograph system (HP 5890–5972 MS, Agilent Technology Inc., Santa Clara, CA, USA) equipped with an HP-5MS (60 m  ×  0.25 mm  ×  0.25 μm, Agilent Technologies) column, was used for analysis. For phenylacetaldehyde, the oven program was started at 50 °C for 2 min, ramped with a rate of 10 °C/min to 180 °C, then, to 280 °C, with a rate of 25 °C/min and held for 5 min. The MS detector temperature was 180 °C and the transfer line temperature was 280 °C. Selective ion monitoring (SIM) mode (65, 91, 92, 120) was used to quantify PheAce. The n-decanal was used as the internal standard (IS) and was detected with ions (57, 70, 82, 95, 112). The LOD and LOQ of PheAce were 0.007 μg/g and 0.02 μg/g. The concentration of PheAce in the sample was calculated with the standard curve equation of PheAce/IS by injecting different concentrations of PheAce and fixed amounts of IS. For volatile compounds, the analyte was desorbed from the SPME fiber at the injector port at 280 °C for 5 min in a splitless mode. The carrier gas (flow rate of 1 mL/min) was high-purity helium. The oven temperature program was set as follows: 40 °C for 2 min, increased (rate of 3 °C/min) to 80 °C, increased (rate of 5 °C/min) again to 150 °C, and finally to 250 °C (at a rate of 10 °C/min) and held for 1 min. The MS detector temperature was 150 °C and the transfer line temperature was 250 °C. The 3 min solvent delay was used to process MS data with total ion scanning in the 40 to 550 *m*/*z* mass range (rate of 1 scan/s). NIST 2008 Mass Spectral Library was used to identify the volatile compounds.

### 2.9. Color Measurement

CIELAB parameters (L*, a*, and b*) of the fried chicken breast were measured with a HunterLab MiniScan EZ 4500 L (Hunter Associates Laboratory Inc., Reston, VA, USA). The colorimeter was calibrated by white and black plates. The ΔE showed the color difference of the PS-coated chicken compared to the control chicken with no coating according to the equation:

ΔE = √(〖ΔL〗^2^ +〖Δa〗^2^ +〖Δb〗^2^) (1)

where ΔL, Δa, and Δb were the differences between the color coordinates of the chicken breasts dipped, the PS coating, and the chicken breasts with no coating.

### 2.10. Cooking Loss Percentage

The difference between the weights of chicken breasts before and after frying was used to calculate the cooking loss percentage according to:

Cooking Loss percentage = (Wb − Wa/Wb) × 100(2)

where Wb and Wa were the weights before and after frying, respectively.

### 2.11. Tenderness

A texture analyzer (TA-XT2, Texture Technologies Corp, Stable Micro Systems, Surrey, UK) coupled with a Meullenet–Owens razor shear blade (MORS) was used for tenderness analysis of PS-coated and controlled chicken breasts. The crosshead speed of 10 mm/s and the blade penetration depth of 15 mm were set [19]. The fried chicken breast cuts were put in a vertical direction to the orientation of muscle fiber under the razor shear blade [20]. Shear force (maximum force (N)) and shear energy (area under maximum force vs. displacement curve (N.mm)) were calculated by the texture analyzer software (Version: 6.1.16.0).

### 2.12. Texture Profile Analysis (TPA)

Texture profile analysis was performed as suggested by [21]. The sample was cut into 2 × 2 × 2 cm diameter pieces using a sharp knife. The TPA was conducted by a TAXT2i texture analyzer (Stable Micro Systems, Godalming, UK) with a 30 kg load cell. A 50 mm cylindrical flat probe and double bite compression method (with a 2 s rest period between the two bites) were used. The samples were compressed to 80% (1.6 cm compression) of their original height (20 cm) by the probe with a crosshead speed of 0.8 mm/s. The TPA curve of force (N) v/s time (S) was plotted and used to derive the instrumental texture attributes such as hardness, cohesiveness, springiness, and chewiness. Hardness (N) was noted as the maximum force during the first compression cycle. Springiness was calculated as the height recovered after the first compression. Cohesiveness was reported as the ratio of positive force area under the second and first compression cycles, and chewiness was calculated by multiplying gumminess and springiness [21].

### 2.13. Statistical Analysis

The SAS 9.4 software was used to determine whether there was a significant difference (*p* < 0.05) between samples at a 95% confidence level. Experiments were conducted in triplicates. For texture, PhIP, and PheAce analysis, six measurements were conducted per sample to account for variations in measurements. Analyses were carried out with the PROC GLIMMIX procedure with Tukey’s test. Different letters in each column on the tables show significant differences.

## 3. Results

### 3.1. Effect of Chicken Breast Immersion Time in PS-Lys Coating on PhIP Level and Possible Mechanism for PhIP Reduction

The results of the PhIP reduction are listed in Table 1. Results showed that PS-0.25% Lys-coated chicken had a significantly (*p* < 0.05) lower PhIP level compared to the control chicken without coating. The control chicken had an average PhIP level of 97.10 ng/g and with treatments, the level was decreased to an average of 18.79–34.76 ng/g. The average PhIP level for 15 min and 30 min dipped chicken was 24.01 ng/g and 26.14 ng/g, respectively, with no significant differences between them. For 1 h and 3 h dipped chicken, the PhIP level was 33.51 ng/g and 34.49 ng/g, respectively with no significant difference (*p* > 0.05), and after 6 h of immersion, the PhIP level decreased to 18.79 ng/g. However, there were no significant differences (*p* > 0.05) between the PhIP levels of 15 min, 30 min, and 6 h dipped chicken. Therefore, with 15 min of immersion of chicken in a PS-Lys coating, the PhIP level decreased to a level comparable to the 6 h immersion. However, in an earlier study [22] chicken marinated with red wine for 24 h had significantly (*p* < 0.05) lower PhIP levels (8.8–12 ng/g) compared to treatments with 30 min and 3 h of marination time. This was attributed to antioxidant compounds and AAs such as proline which are present in the wine and may be effective at a longer treatment time. Moreover, in another study in which beef was marinated with green tea, the PhIP level was decreased by increasing the marinating time up to 6 h. Pearson correlation coefficient showed that the PhIP reduction in marinated meat was highly significant with the increase in marinating time [23]. This can be attributed to the more complex composition of the marinating compound in the mentioned studies compared to the current study in which only Lys is incorporated with the PS solution. However, with 15 min of immersion of chicken breast in this coating solution, the PhIP level could be decreased to a concentration that can be obtained with a longer marinating time by green tea and wine. Lys was previously shown to decrease the PhIP either by direct addition to beef patties [24] or by incorporation in edible films applied to chicken surfaces 15 min prior to frying [12]. The exact mechanism of PhIP prevention by amino acids is still unclear; however, some proposed mechanisms in the literature include scavenging of the PhIP intermediates. One possible mechanism for the prevention of high levels of PhIP was stated to be the PheAce scavenging capability of a compound [24,25]. Therefore, we aimed to study the PheAce content of the chicken with no coating and chicken dipped in the PS-25% Lys coating solution. Results of GC-MS analysis of PheAce showed that the PheAce content in coated chicken breast (0.09 ng/g chicken crust) was significantly (*p* < 0.05) higher than that of the uncoated chicken breast (0.03 ng/g chicken crust). However, results of previous studies showed the reduction of PheAce in the model system included non-precursor amino acids. In a model system containing all the precursors for PhIP formation and non-precursor amino acids at different concentrations, the PheAce concentration was significantly lower in the model systems containing 0.125% to 2% of several AAs including Lys [24]. Another study showed that benzaldehyde and phenylacetaldehyde are key intermediates for the formation of PhIP. This indicated that histidine–proline combinations are capable of scavenging phenylacetaldehyde and benzaldehyde simultaneously, which increases the suppression of PhIP formation [13]. In the current study, benzaldehyde concentration was not studied alone; however, the results from the volatile compound analysis by GC-MS, which was conducted on chicken crust with the SPME method, showed that there were no significant differences (*p* > 0.05) between the benzaldehyde contents of chicken breast crust with and without the PS-0.25% Lys coating. This phenomenon can be attributed to the complex PhIP formation pathways and the intermediates. To inhibit the PhIP formation, all these pathways and intermediates should be blocked. However, there is no information about other potential key ketone or aldehyde intermediates which could be responsible for generating PhIP and whether blocking these intermediates could reduce PhIP [13]. Moreover, the mentioned studies were conducted on the model systems; therefore, this inconsistency in results can be attributed to the complexity of real food (chicken) and the interactions due to the matrix effect.

### 3.2. Volatile Compounds in Chicken Breast Dipped in PS-0.25% Lys Coating Solution versus Uncoated Chicken

**Figure 1:**
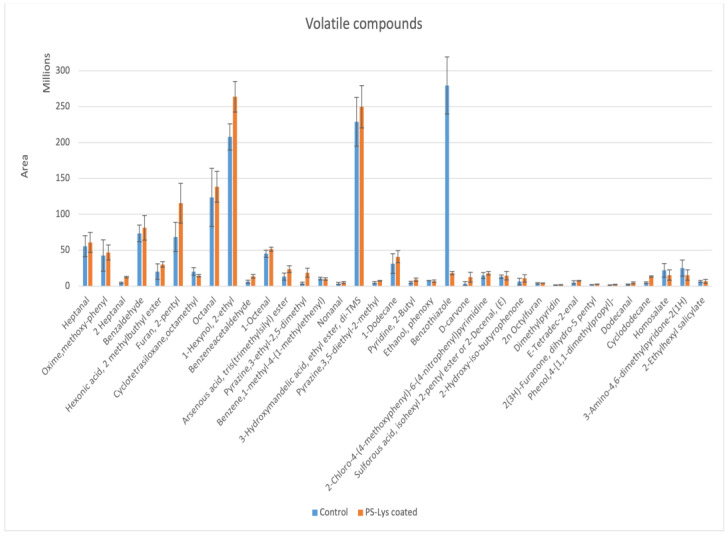
Volatile compounds of fried chicken breast crust with potato starch-0.25% lysine coating versus without coating.

Chicken breast dipped in PS-0.25% Lys for 15 min and chicken breast with no coating were selected to study the volatile compounds. Different volatile compounds were released from chicken during cooking mainly through the Maillard reaction, lipid oxidation, and degradation, as well as in the interaction between lipid-oxidation products with Maillard intermediates [26]. As Figure 1 illustrates, the differences in the volatile compound profile of fried chicken crust (195 °C/7.5 min) dipped in a PS-0.25% Lys coating solution and the control chicken crust without coating were characterized with GC-MS (their GC-MS chromatogram and a table of retention times and integrated data are presented in the Supplementary Materials Table S1 and Figure S1). Volatile compounds identified in fried chicken crusts by the SPME-GC-MS method included nine aldehydes (nonanal, 1-octenal, octanal, heptanal, 2-heptenal, dodecanal, 2-decenal, benzaldehyde, and phenylacetaldehyde), 2-pyrazines (3,5-diethyl-2-methylpyrazine, 3-ethyl-2,5-dimethylpyrzines), 1 alcohols (phenoxy ethanol), 1 sulfur compound (benzothiazole), and 3 furan (2-pentyl furan, 2n octylfuran, dihydro-5-2(3H)-Furanone), 1 hydrocarbon (1-methyl-4-(1-methylethenyl)-benzene, 1 Ketone (D-carvone), 3 pyridines (2-Butyl pyridine and dimethylpyridine), 3 esters (2-methyl butyl ester-hexanoic acid, 3-hydroxymandelic acid ethyl ester, di-tms, arsenous acid, tris(trimethylsilyl) ester), and a compound named (2-Hydroxy-iso-butyrophenone) were identified in fried the chicken crusts. The most abundant volatile compounds were benzothiazole, 3-hydroxymandelic acid ethyl ester, and di-tms, followed by benzaldehyde and other mentioned compounds. As shown in Figure 1, the PS-0.25% Lys-coated chicken crust had significantly higher (*p* < 0.05) levels of 2-heptenal, PheAce, D-carvone, dodecanal, and cyclododecane; however, the treatment had a significantly (*p* < 0.05) lower benzothiazole level. No significant differences (*p* > 0.05) were shown in the levels of other volatile compounds between the control and coated chicken.

Immersion of the chicken in a PS-0.25% Lys coating solution significantly enhanced (*p* < 0.05) the formation of the roast odor 3-ethyl-2,5-dimethylpyrazine, and 3,5-diethyl-2-methylpyrazine after frying, compared to controls. Therefore, findings suggested that immersion in a PS-0.25% Lys coating solution can promote the formation of roasted and nutty odors in fried chicken. Pyrazines, furan, and thioethers were detected in the Maillard reaction products which contributed to the roasted, meaty, and coffee odors [27]. Benzothiazole was reported to be the major nitrogen- and/or sulfur-containing compound in chicken byproducts [28]. Thiazoles were shown to be present in grilled, fried, or roasted meats, in high amounts. These compounds have low odor threshold values and therefore, are considered important contributors to meat-like aroma [29]. The significant increase in PheAce (green and floral odor) indicated by the SPME-GC-MS study of volatile compounds compared to the control confirmed the result from a splitless injection of the chicken crust extract to GC-MS in the Section 3.1. This indicates that the PS-Lys coating was involved in the Maillard reaction during cooking and influenced the formation of different Strecker aldehydes. However, researchers [24] have shown that the addition of AAs including Lys could significantly decrease the PheAce level in a beef model system. These inconsistencies in the results could be due to the more complex matrix of real chicken compared to the model system. Moreover, the coating did not significantly (*p* > 0.05) affect the formation of alcohols and furans, which are mainly produced from Maillard reactions or lipid oxidation and are responsible for unpleasant odors [30,31]. Various volatiles such as aliphatic hydrocarbons, alcohols, aldehydes, ketones, esters, carboxylic acids, and some aromatic hydrocarbons are formed through lipid degradation (such as fatty acid oxidation of lipids). Moreover, although there are high levels of ester compounds in the chicken crusts, they mostly do not affect the odor of meat products, except for lactone and thioester [29].

### 3.3. Effect of Immersion in PS-0.25% Lys Coating Solution on Physical Characteristics of Chicken Breasts

#### 3.3.1. Cooking Loss Percentage

The results for the cooking loss percentages of chicken breasts are presented in Table 2. The average cooking loss percentages were in the range of 45.75% to 53.34% and there were no significant differences between the values, showing that the immersion time in PS-0.25% Lys coating solution did not have a significant effect on cooking loss percentages of chicken breasts and the control chicken without coating. However, in another study, coating the three-dimensional printed crab samples with 12% PS gel decreased the cooking loss after frying, which can be attributed to a higher concentration of PS (12%) compared to the current study (5%), which shows better protection against moisture loss [32].

#### 3.3.2. Color

Color parameters are listed in Table 3. The Maillard reaction occurs during the frying process due to sugar–protein interactions, lipid oxidation reactions, and the formation of primary and secondary oxidation products. This reaction can cause color changes in the fried samples [33]. Chicken dipped in the coating solution versus control chicken with no coating had ∆E (differences in color parameters) in the range of 3.45–6.50 with no significant differences (*p* > 0.05) between them. Therefore, immersion of chicken breast in a PS-0.25% Lys coating solution for different times does not affect the surface color after frying because the change in color parameters was not significant or visible to humans [34].

#### 3.3.3. Tenderness and Texture Profile Analysis (TPA)

Table 4 shows the shear force and shear energy of chicken breasts. These parameters can be an estimation of the tenderness of the chicken. The average sheer force of the chicken samples was in the range of 8.32–12.10 N and shear energy was in the range of 34.3–53.06 N.mm. Compared with the control, samples of chicken with different immersion times in a PS-0.25% Lys coating showed no significant differences (*p* < 0.05) in values for shear force and shear energy. Texture profile analysis (Table 5) was used to compare uncoated control samples with chicken breasts dipped in the PS-0.25% Lys coating at different times. There were no significant differences (*p* > 0.05) in values for cohesiveness, springiness (%), and chewiness. However, compared to the control, the hardness value was significantly higher in 6 h dipped chicken breast with an average hardness of 271 N. In an earlier study, coated chicken with starch nanocrystals had higher hardness compared to the control before frying [32]. There were no significant differences (*p* < 0.05) between the hardness of chicken breasts dipped in PS-0.25% Lys at different times. The higher hardness was attributed to the higher moisture loss of the samples during the frying process [32]

## 4. Conclusions

This study has shown that immersing chicken breasts in potato starch (PS) coating solution with 0.25% Lys for just 15 min achieves a PhIP level comparable to that obtained after a 6-h immersion. Furthermore, no significant differences were observed in cooking loss percentage, texture properties, and color parameters between coated and uncoated chicken. Interestingly, the significantly lower levels of phenylacetaldehyde in uncoated chicken compared to 0.25% Lys-PS coated chicken (15 min) suggest an alternative pathway through which this coating may mitigate PhIP levels. Volatile compound analysis has revealed increased levels of specific aroma compounds, such as pyrazines, in coated chickens compared to uncoated ones. These findings contribute valuable insights into the potential benefits of the PS-Lys coating in reducing PhIP while maintaining the physicochemical properties of the pan-fried chicken breasts.

## Figures and Tables

**Table 1:** PhIP concentration and PhIP percent reduction results of chicken breasts dipped in PS-0.25% lysine coating solution versus chicken without coating. Values (means ± SD from Excel, *n* = 3) in the same column followed by different uppercase letters indicate significant differences (*p* < 0.05). Lys: lysine, PS: potato starch, Cons: concentration, PhIP: 2-amino-1-methyl-6-phenylimidazo [4,5-b] pyridine.

Chicken Breast	Sample Treatment Time	PhIP Cons (ng/g)	PhIP Percent Reduction (%)
Control	0	97.10 ± 5.91 ^A^	-
PS-0.25%-Lys coated	15 min	24.01 ± 5.57 ^D^	75.46 ± 5.45 ^BA^
	30 min	26.14 ± 5.77 ^CD^	74.41 ± 5.65 ^B^
	1 h	33.51 ± 2.75 ^CB^	65.96 ± 1.23 ^C^
	3 h	34.49 ± 1.48 ^B^	66.23 ± 1.45 ^C^
	6 h	18.79 ± 3.62 ^D^	81.61 ± 3.55 ^A^

**Table 2:** The cooking loss percentage of chicken breasts dipped in PS-0.25% lysine coating solution versus the chicken without coating. Values (means ± SD from Excel, *n* = 3) in the same column followed by different uppercase letters indicate significant differences (*p* < 0.05). Lys: lysine, PS: potato starch.

Chicken Breast	Sample Treatment Time	Cooking Loss (%)
Control	0	47.47 ± 2.84 ^A^
PS-0.25%-Lys coated	15 min	47.74 ± 5.94 ^A^
	30 min	53.34 ± 3.81 ^A^
	1 h	47.39 ± 2.04 ^A^
	3 h	45.75 ± 4.08 ^A^
	6 h	48.57 ± 3.55 ^A^

**Table 3:** Color parameters of chicken breasts dipped in PS-0.25% lysine coating solution versus the chicken without coating. Values (means ± SD from Excel, *n* = 3) in the same column followed by different uppercase letters indicate significant differences (*p* < 0.05). Lys: lysine, PS: potato starch, L*: lightness, a*: redness, b*: yellowness, ∆E: difference in color parameters compared to the control.

Chicken Breast	Sample Treatment Time	L*	a*	b*	∆E
Control	0	42.15 ± 1.34 ^A^	20.50 ± 0.5 ^A^	28.77 ± 0.97 ^A^	-
PS-0.25% Lys coated	15 min	41.34 ± 3.33 ^A^	18.89 ± 0.86 ^A^	26.70 ± 1.03 ^A^	3.45 ± 0.20 ^A^
	30 min	39.76 ± 2.60 ^A^	21.53 ± 1.70 ^A^	28.09 ± 2.04 ^A^	6.50 ± 2.44 ^A^
	1 h	40.58 ± 0.70 ^A^	21.24 ± 2.20 ^A^	28.37 ± 2.27 ^A^	3.55 ± 0.59 ^A^
	3 h	40.65 ± 2.45 ^A^	21.33 ± 2.53 ^A^	28.08 ± 1.75 ^A^	4.22 ± 3.03 ^A^
	6 h	40.77 ± 3.45 ^A^	20.36 ± 3.15 ^A^	27.44 ± 2.63 ^A^	5.20 ± 1.85 ^A^

**Table 4:** Shear force and shear energy values of chicken breasts dipped in PS-0.25% lysine coating solution versus the chicken without coating. Values (means ± SD from Excel, *n* = 3) in the same column followed by different uppercase letters indicate significant differences (*p* < 0.05). Lys: lysine, PS: potato starch.

Chicken Breast	Sample Treatment Time	Shear Force (N)	Shear Energy (N.mm)
Control	0	12.10 ± 0.85 ^A^	43.10 ± 10.35 ^A^
PS-0.25% Lys coated	15 min	10.74 ± 2.60 ^A^	53.06 ± 7.90 ^A^
	30 min	10.79 ± 2.08 ^A^	34.3 ± 9.82 ^A^
	1 h	10.77 ± 43.05 ^A^	43.05 ± 16.09 ^A^
	3 h	8.32 ± 2.57 ^A^	47.25 ± 18.62 ^A^
	6 h	10.46 ± 1.09 ^A^	55.46 ± 12.40 ^A^

**Table 5:** The texture profile of chicken breasts dipped in PS-0.25% lysine coating solution versus the chicken without coating. Values (means ± SD from Excel, *n* = 3) in the same column followed by different uppercase letters indicate significant differences (*p* < 0.05). Lys: lysine, PS: potato starch.

Chicken Breast	Sample Treatment Time	Hardness (N)	Cohesiveness	Springiness (%)	Chewiness
Control	0	184.05 ± 16.55 ^B^	0.41 ± 1.93 ^A^	57.04 ± 0.05 ^A^	49.29 ± 4.83 ^A^
PS-0.25% Lys coated	15 min	245.78 ± 11.08 ^BA^	0.46 ± 0.05 ^A^	60.11 ± 8.80 ^A^	65.56 ± 5.66 ^A^
	30 min	231.17 ± 41.08 ^BA^	0.46 ± 2.23 ^A^	58.375 ± 0.05 ^A^	62.03 ± 14.15 ^A^
	1 h	243.27 ± 27.65 ^BA^	0.46 ± 2.18 ^A^	58.542 ± 0.04 ^A^	67.54 ± 10.89 ^A^
	3 h	249.40 ± 33.12 ^BA^	0.44 ± 2.37 ^A^	52.477 ± 0.06 ^A^	61.13 ± 9.69 ^A^
	6 h	257.10 ± 25.26 ^A^	0.45 ± 3.16 ^A^	59.458 ± 0.06 ^A^	64.78 ± 13.48 ^A^

## Data Availability

Data is contained within the article or Supplementary Materials.

## Supplementary Materials

The following supporting information can be downloaded at: https://www.mdpi.com/article/10.3390/foods13020222/s1, Figure S1: A sample chromatogram of volatile compounds from pan-fried chicken crust using SPME-GCMS on the HP-5 column. The carrier gas (flow rate of 1 mL/min) was helium. The oven temperature program was set as follows: 40 °C for 2 min, increased (rate of 3 °C/min) to 80 °C, and increased (rate of 5 °C/min) again to 150 °C, and finally to 250 °C (at a rate of 10 °C/min) and held for 1 min. The MS detector temperature was 150 °C and transfer line temperatures was 250 °C. A 3 min solvent delay was used to process MS data with total ion scanning in the 40 to 550 *m*/*z* mass range (rate of 1 scan/s); Table S1: Volatile compounds from pan-fried chicken crust without coating and PS-0.25% Lys coated chicken crust using the SPME-GCMS method. Qual is the match factor of compounds to the NIST mass spectral library.
