# Supplementary material for: Effect of Immersion Time of Chicken Breast in Potato Starch Coating Containing Lysine on PhIP Levels
Source: Foods. 2024 Jan 10;13(2):222. doi: 10.3390/foods13020222 (PMC10814811; doi:10.3390/foods13020222)
Supplement: Supplementary file 1 [file foods-13-00222-s001.zip › foods-2771457-supplementary.pdf]

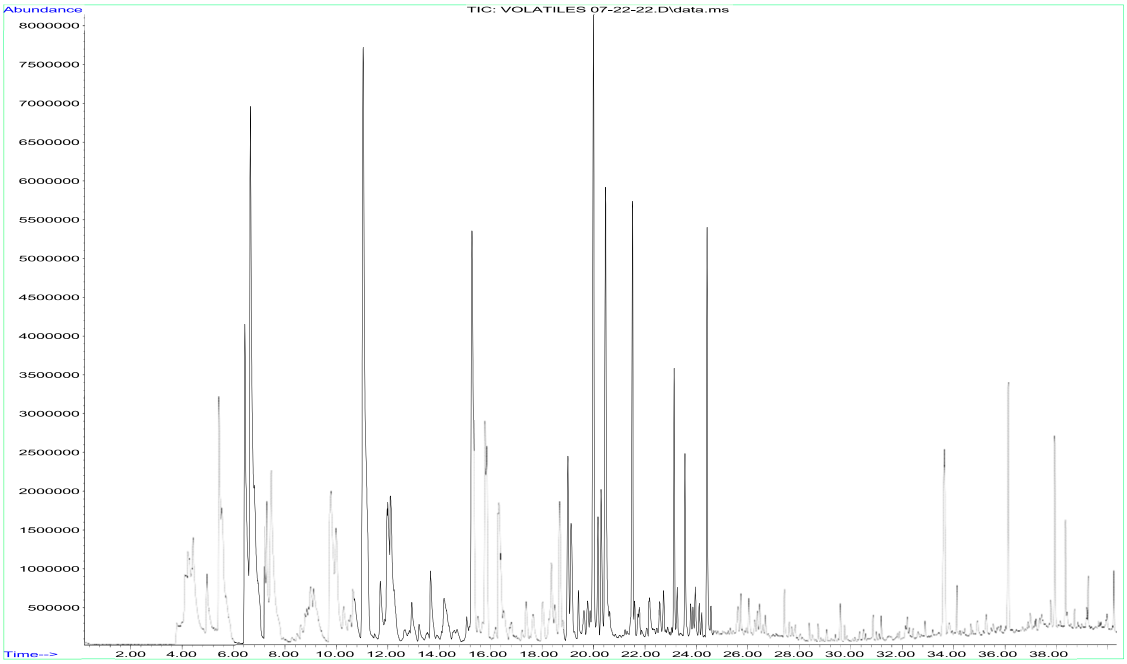


**Supplemental Figure S1** A sample chromatogram of volatile compounds from pan-fried chicken crust using SPME-GCMS on the HP-5 column. The carrier gas (flow rate of 1 mL/min) was helium. The oven temperature program was set as follows: 40 °C for 2 min, increased (rate of 3 °C/min) to 80 °C, and increased (rate of 5 °C/min) again to 150 °C, and finally to 250 °C (at a rate of 10 °C/min) and held for 1 min. The MS detector temperature was 150 °C and transfer line temperatures was 250 °C. A 3 min solvent delay was used to process MS data with total ion scanning in the 40 to 550 m/z mass range (rate of 1 scan/s).

**Supplemental Table S1** Volatile compounds from pan-fried chicken crust without coating and PS-0.25% Lys coated chicken crust using the SPME-GCMS method. Qual is the match factor of compounds to the NIST mass spectral library.

| Volatile compounds | Control (average peak area) | Ps-0.25% Lys coated (average peak area) | Time (min) | Qual |
|---|---|---|---|---|
| Pentanal | 51781425 | 53474012 | 7.467 | 80 |
| 3-methyl Butanal | 20098051.7 | 12203032.67 | 6.43 | 91 |
| 2-methyl Butanal | 17703579.7 | 5398726.667 | 6.62 | 90 |
| 1-Pentanol | 23698309 | 27497310.33 | 9.7 | 72 |
| 2,5-dimethyl pyrazine | 228950532 | 249946235.7 | 12.56 | 92 |
| Heptanal | 55464782 | 60882770 | 15.82 | 97 |
| Oxime,methoxy-phenyl | 42487753.3 | 46738575.67 | 16.293 | 91 |
| 2 Heptenal | 4366712.33 | 12665744.67 | 18.447 | 52 |
| Benzaldehyde | 73237767.7 | 81355280.33 | 18.727 | 97 |
| Hexanal | 277395158 | 334718448.3 | 11.11 | 95 |
| Hexonic acid, 2 methylbuthyl ester | 20194211 | 30073095.67 | 19.641 | 56 |
| Furan, 2-pentyl | 61791911.3 | 115460236.3 | 20.037 | 94 |
| Cyclotetrasiloxane,octamethyl | 23632129.3 | 14351688.67 | 20.191 | 91 |
| Octanal | 123623322 | 138517546 | 20.499 | 98 |
| 1-Hexynol, 2-ethyl | 207873558 | 263782738 | 21.609 | 64 |
| Phenylacetaldehyde | 6072441.67 | 13699854.33 | 22.255 | 93 |
| 1-Octanol | 45112041.3 | 51532644.33 | 23.163 | 87 |
| Arsenous acid, tris(trimethylsilyl) ester | 13112580 | 23608368 | 23.425 | 64 |
| Pyrazine,3-ethyl-2,5-dimethyl | 3699352.67 | 18753311.33 | 23.582 | 83 |
| Benzene,1-methyl-4-(1-methylethenyl) | 10540661.7 | 9821596 | 23.996 | 96 |
| Nonanal | 3582389 | 5082313.667 | 24.267 | 91 |
| 3-Hydroxymandelic acid, ethyl ester, di-TMS | 245617199 | 249946235.7 | 24.595 | 53 |
| Pyrazine,3,5-diethyl-2-methyl | 4685218 | 7314181 | 26.389 | 41 |
| 1-Dodecane | 31315966.7 | 40788765.33 | 27.39 | 96 |
| Pyridine, 2-Butyl | 5254395.67 | 9039639 | 27.728 | 87 |
| Ethanol, phenoxy | 7671781 | 6924037.667 | 28.496 | 90 |
| Benzothiazole | 246087153 | 18157997.33 | 28.805 | 91 |
| D-Carvone | 3538495.33 | 12392784.67 | 29.276 | 96 |
| 2-Chloro-4-(4-methoxyphenyl)-6-(4- nitrophenyl) pyrimidine | 14739506 | 17882631.5 | 29.428 | 43 |
| Sulforous acid, isohexyl 2-pentyl ester or 2-Decenal, (E) | 13141281.3 | 14451404.67 | 29.626 | 86 |
| 2-Hydroxy-iso-butyrophenone | 6400929.33 | 10818169.67 | 30.394 | 56 |
| 2n Octylfuran | 3825391.33 | 4108139.667 | 30.522 | 93 |
| Dimethylpyridin | 1394319 | 1714872.5 | 31.828 | 30 |
| E-Tetradec-2-enal | 5098933.67 | 7487143 | 32.216 | 91 |
| 2(3H)-Furanone, dihydro-5 pentyl | 1845947.67 | 2659569.667 | 32.292 | 72 |
| Phenol,4-[1,1-dimethylpropyl]- | 1084755.67 | 2054120 | 33.08 | 70 |
| Dodecanal | 2067531 | 4475783.667 | 33.145 | 95 |
| Cyclododecane | 4661380 | 13438220.33 | 35.983 | 94 |
| Homosalate | 21939242.3 | 15354381 | 40.23 | 83 |
| 3-Amino-4,6-dimethylpyridine-2(1H) | 25114866.5 | 15354381 | 40.23 | 43 |

| | | | | |
|---|---|---|---|---|
| 2-Ethylhexyl salicylate | 6497587 | 6714596 | 39.189 | 99 |
